# Author Correction: The evolution of RET inhibitor resistance in RET-driven lung and thyroid cancers

**DOI:** 10.1038/s41467-022-29700-y

**Published:** 2022-04-05

**Authors:** Ezra Y. Rosen, Helen H. Won, Youyun Zheng, Emiliano Cocco, Duygu Selcuklu, Yixiao Gong, Noah D. Friedman, Ino de Bruijn, Onur Sumer, Craig M. Bielski, Casey Savin, Caitlin Bourque, Christina Falcon, Nikeysha Clarke, Xiaohong Jing, Fanli Meng, Catherine Zimel, Sophie Shifman, Srushti Kittane, Fan Wu, Marc Ladanyi, Kevin Ebata, Jennifer Kherani, Barbara J. Brandhuber, James Fagin, Eric J. Sherman, Natasha Rekhtman, Michael F. Berger, Maurizio Scaltriti, David M. Hyman, Barry S. Taylor, Alexander Drilon

**Affiliations:** 1 Department of Medicine, Memorial Sloan Kettering Cancer Center, New York, NY USA; 2 Marie-Josee and Henry R. Kravis Center for Molecular Oncology, Memorial Sloan Kettering Cancer Center, New York, NY USA; 3 Department of Pathology, Memorial Sloan Kettering Cancer Center, New York, NY USA; 4 Department of Human Oncology and Pathogenesis Program, Memorial Sloan Kettering Cancer Center, New York, NY USA; 5 University of Miami, Miller School of Medicine, Department of Biochemistry and Molecular Biology/Sylvester Comprehensive Cancer Center, Miami, FL USA; 6 Department of Epidemiology and Biostatistics, Memorial Sloan Kettering Cancer Center, New York, NY USA; 7 Weill Cornell Medical College, New York, NY USA; 8 Present Address: Loxo Oncology at Lilly, Stamford, CT USA; 9 Present Address: AstraZeneca, Waltham, MA USA

**Keywords:** Oncogenes, Cancer genomics, Cancer therapeutic resistance

Correction to: *Nature Communications* 10.1038/s41467-022-28848-x, published online 18 March 2022.

In this article the author name Srushti Kittane was incorrectly written as Srusthi Kittane. The original article has been corrected.

