# Supplementary material for: The evolution of RET inhibitor resistance in RET-driven lung and thyroid cancers
Source: Nat Commun. 2022 Mar 18;13:1450. doi: 10.1038/s41467-022-28848-x (PMC8933489; doi:10.1038/s41467-022-28848-x)
Supplement: Supplementary file 1 — Supplementary Information [file 41467_2022_28848_MOESM1_ESM.pdf]

Supplementary Information

Supplemental Figures

Supplemental Fig. 1: Consort diagram for the study cohort.

Supplemental Fig. 2: Outcomes based on treatment status at enrollment.

Supplemental Fig. 3: Study and contemporary cohort alteration frequencies.

Supplemental Fig. 4: Whole-exome sequencing results.

Supplemental Fig. 5: Plasma detection and response.

Supplemental Fig. 6: Pre-treatment tissue and plasma concordance.

Supplemental Fig. 7: Spectrum of PI3K alterations and response.

Supplemental Fig. 8: Mutant KRAS induces pERK in *RET* M918T-mutant thyroid cancer cells.

Supplemental Fig. 9: Pre- and post-treatment IHC of LCNEC case.

Supplemental Tables

Supplemental Table 1: Clinical and demographic summary of the study cohort.

Supplemental Table 2: Patient-level clinical and molecular data.

Supplemental Table 3: Whole-exome sequencing.

Supplemental Table 4: Annotation of prior MKI response.

Supplemental Table 5: Paired pre- and post-progression tissue and plasma results

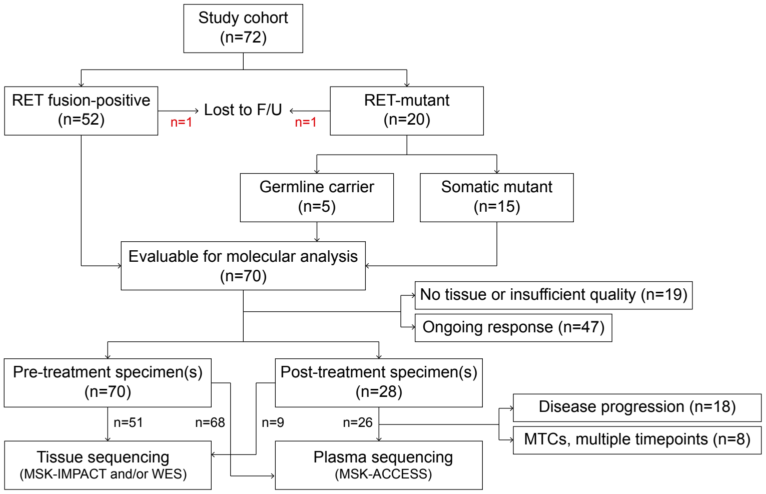

Supplemental Fig. 1: Consort diagram for the study cohort. A diagrammatic representation of the 72 treated patients and the degree and type of their molecular profiling.

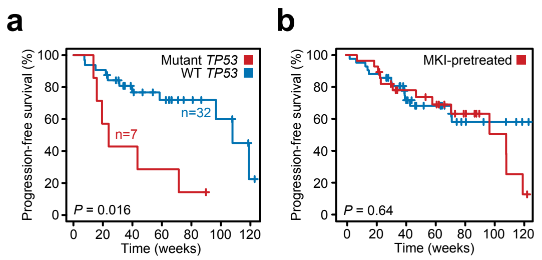

Supplemental Fig. 2: Outcomes based on treatment status at enrollment. a) The clinical outcome of patients treated with selpercatinib therapy based on whether their tumors possessed *TP53* mutations (red and, respectively) detected by pre-treatment tumor tissue sequencing (shown are those patients with *RET* alterations shedding in baseline plasma, multivariable model including *RET* shedding as a covariate: HR = 3.5, 95% CI 1.3-9.7, *P* = 0.016, likelihood ratio test). **b)** As in panel (a), but in patients receiving MKI therapy prior to selpercatinib treatment or not (HR = 1.2, 95% CI 0.6-2.6, *P* = 0.64, likelihood ratio test).

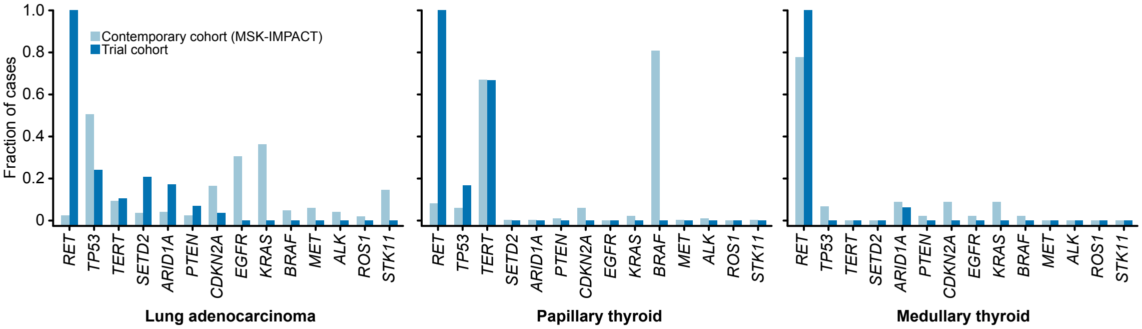

Supplemental Fig. 3: Study and contemporary cohort alteration frequencies. The frequency of alteration of key genes (grouped by cancer type) mutated in pre-treatment tumor specimens of the study cohort (dark blue) are shown compared to their frequency in the contemporary cohort from which they were drawn (prospectively sequenced MSK-IMPACT cohort, light blue).

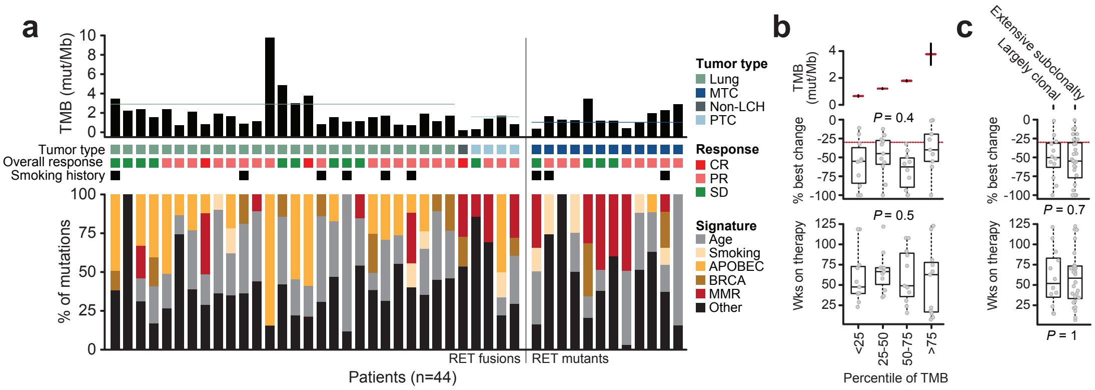

Supplemental Fig. 4: Whole-exome sequencing results. a) From whole-exome sequencing of pre-treatment tumor specimens in 44 cases, the tumor mutational burden (TMB) is shown (top) as are the mutational signatures inferred from the somatic mutations of each case (bottom, see legend at right). **b)** The percent best response and weeks on selpercatinib therapy across the spectrum of TMB (top, mean and 95% CI of TMB of cases grouped as percentiles as indicated at bottom, N=11 each quartile of 44 patients). **c)** The percent of patients with clinical benefit from selpercatinib therapy as a function of the degree of intratumoral heterogeneity among somatic mutations detected in the whole-exome sequencing of pre-treatment tumors. *P* values as indicated, one-way ANOVA and Wilcoxon test, N=14 Largely clonal, N=30 Extensive subclonality, N=44 total patients.

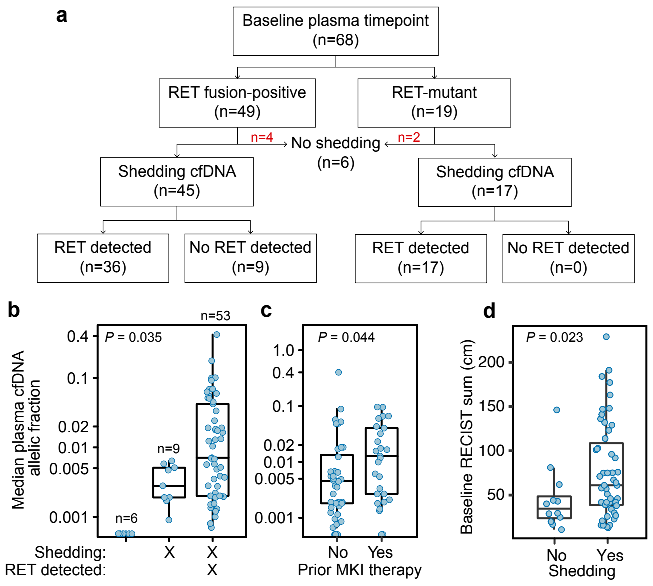

Supplemental Fig. 5: Plasma detection and response. a) The breakdown of plasma cfDNA findings at baseline as a function of RET alteration type. For the purposes of this breakdown, patients were considered as shedding cfDNA into circulation if sequencing of the baseline time point identified one or more somatic mutations. The median allelic fraction of somatic mutations identified in the baseline plasma specimen for each patient based on **b)** their shedding status and whether the enrolling RET alteration was detected; (*Ps* as indicated, Wilcoxon test, N=68 patients) or **c)** whether they had received prior MKI therapy (*Ps* as indicated, Wilcoxon test, N=68 patients). **d)** Shedding status versus sum of RECIST assessment as a surrogate for disease burden at baseline (*Ps* as indicated, Wilcoxon test, N=62 patients).

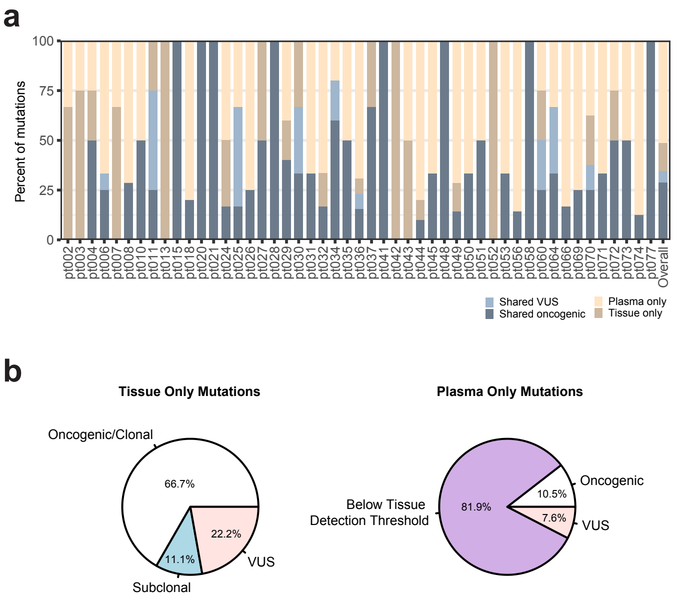

Supplemental Fig. 6: Pre-treatment tissue and plasma sequencing concordance. a) In total, 49 patients with paired pre-treatment tissue and baseline plasma samples showing the proportion of mutations that were shared or exclusive to the tissue or plasma specimen (legend as indicated). The concordance between tissue and plasma was 84% for the sensitizing *RET* alteration, and 34.7% shared, 13.9% exclusive in tissue, and 51.5% exclusive in plasma overall, with varying concordance across all samples as shown. **b)** Breakdown of the mutations exclusive to the tissue or plasma.

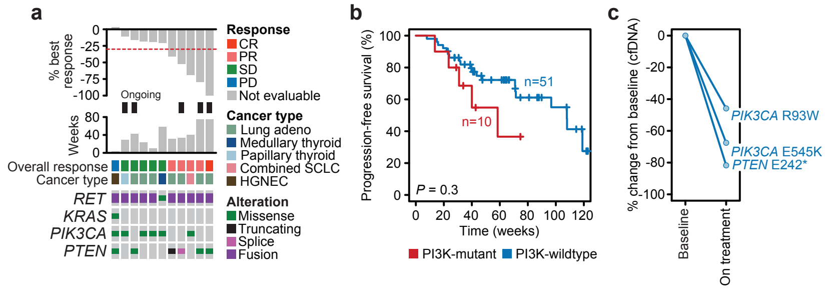

Supplemental Fig. 7: Spectrum of PI3K alterations and response. a) PI3K alterations detected in either tissue or plasma at baseline in 11 patients is shown. From top to bottom: the percent best response, weeks on therapy, and detected mutations in the indicated genes (annotations as in main text Fig. 1D). **b)** The impact of PI3K alterations at baseline on clinical benefit to selpercatinib therapy (multivariable model including shedding, HR=1.7; 95% CI 0.62-4.71; *P* as indicated, likelihood ratio test). Excludes cases with primary resistance due to *KRAS* mutations. **c)** In three patients with PI3K alterations detected at baseline, longitudinal cfDNA specimens, and clinical benefit from selpercatinib therapy, the percent change from baseline is shown for the *PIK3CA* or *PTEN* mutations after treatment initiation.

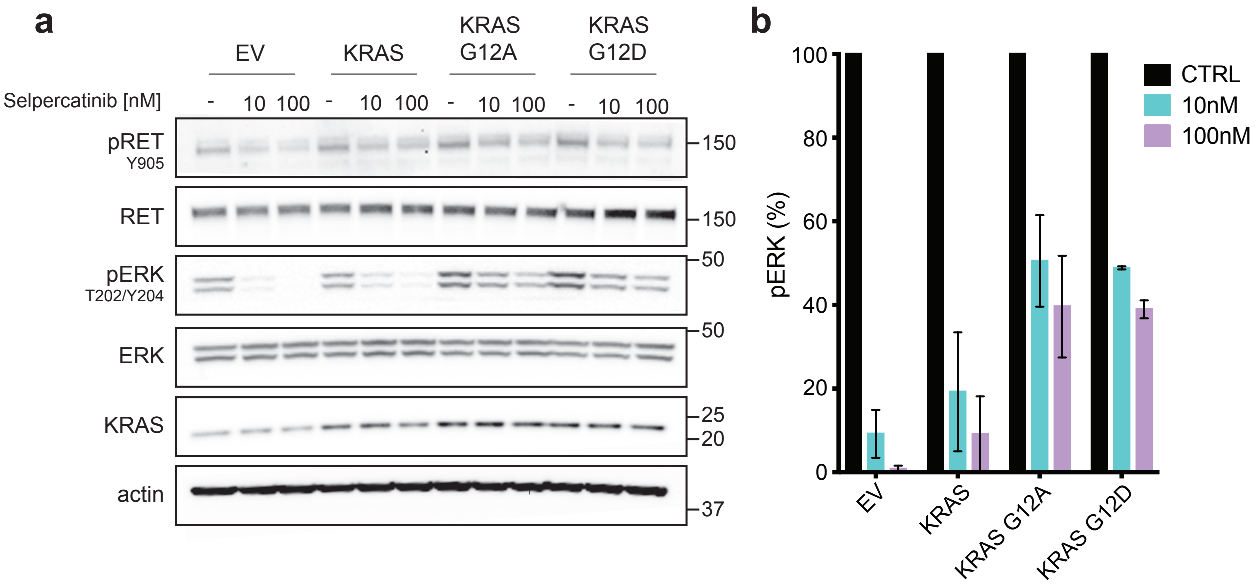

Supplemental Fig. 8: Mutant KRAS induces pERK in RET M918T-mutant thyroid cancer cells. a) Expressing one of multiple KRAS mutations (G12A and G12D) observed arising in patients on selpercatinib treatment in a RET M918T-mutant medullary thyroid cancer cell line MZ-CRC-1 led to increased phosphorylated ERK (pERK) that could not be abrogated by increasing concentrations of selpercatinib. **b)** The quantification by densitometry (n=2 experiments) of pERK levels in MZ-CRC-1 cells from panel A studies.

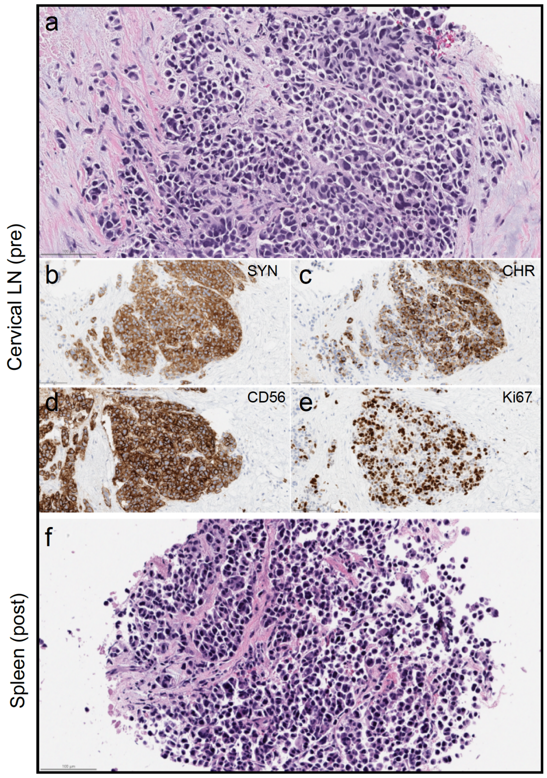

Supplemental Fig. 9: Pre- and post-treatment IHC of LCNEC Case. Pre-treatment biopsy H&E showing neuroendocrine morphology (a), confirmed by staining for synaptophysin, chromogranin, CD56, and Ki67 (b-e) with features consistent with large cell neuroendocrine carcinoma of the lung (LCNEC). Post-treatment biopsy (f) showing similar features and no visible lineage changes.

Supplemental Table 1: Clinical and demographic summary of the study cohort.

Summarized here are the key clinical and demographic features of the 72 selpercatinib-treated patients studied here.

**Median age, years (range)**	62.5 (16-88)
**Sex, n (%)**	
Male	38. (52.7)
Female	34 (47.2)
**Tumor Histology**	
Lung adenocarcinoma	42 (58.3)
Lung adenosquamous	1 (1.3)
LCNEC of the lung	1 (1.3)
Medullary thyroid cancer	20 (27.8)
Papillary thyroid cancer	5 (6.9)
Poorly differentiated thyroid cancer	1 (1.3)
HGNEC of the colon and rectum	1 (1.3)
Non-Langerhans cell histiocytosis	1 (1.3)
**Enrolling *RET* fusion, n (%)**	52 (72.2)
KIF5B	28
CCDC6	14
NCOA4	2
Other	8
**Enrolling *RET* mutation, n (%)**	20 (27.8)
M918T	10
C634R	3
Other	7
**Origin of RET mutation**	
Somatic	15 (75)
Germline	5 (25)
**Median Prior Therapies (range)**	1 (0-7)
**Prior targeted therapies received**	
Cabozantinib	17
Vandetanib	11
RXDX-105	7

Supplemental Table 2: Patient-level clinical and molecular data.

Study_ID	Sample	MSK-IMPACT	EXOME	ACCESS	Tumor_Type	Alteration type	Origin	Enrolling RET lesion	Enrolling RET Lesion Category	Best Overall Response	RECIST_response	Lines Prior to Selpercatini	Prior MKI therapy	Smoking history	Days on Selpercatinib	Days to progression*	Ongoing therapy at time of data analysis	Baseline Extra Alteration	Baseline Extra Alteration Detailed	Acquired Alterations - Tissue	Acquired Alterations - Plasma
EDD_ret_pt001	ACCESS			Longitudinal	Lung Adenocarcinoma	Fusion	Somatic	CCDC6-RET	fusion	PR	-76	2	Yes	No	944	756	Yes				
EDD_ret_pt002	IMPACT_EXOME_ARCHER_ACCESS	Pre	Pre	Baseline	Lung Adenocarcinoma	Fusion	Somatic	KIF5B-RET	fusion	SD	NTL	2	No	No	523		Yes				
EDD_ret_pt003	IMPACT_EXOME_ARCHER_ACCESS	Pre	Pre	Baseline	Lung Adenocarcinoma	Fusion	Somatic	RBPMS-RET	fusion	PR	-87.671	2	No	Yes	580	275	Yes	RET_fusion			
EDD_ret_pt004	IMPACT_EXOME_ACCESS	Pre	Pre	Longitudinal	Papillary Thyroid	Fusion	Somatic	CCDC6-RET	fusion	PR	-32.609	2	No	No	359	304	No				
EDD_ret_pt006	IMPACT_ACCESS	Pre+Post		Longitudinal	Medullary Thyroid	Mutation	Somatic	M918T	KD_oncogenic	SD	-25	3	Yes	Yes	191	163	No	RET_mut	V804M	KRAS G12D	KRAS G12D/G13D, RET Y806C
EDD_ret_pt007	IMPACT_ACCESS	Pre		Baseline	Lung Adenocarcinoma	Fusion	Somatic	EML4-RET	fusion	PR	-53.49	1	Yes	No	615		Yes				
EDD_ret_pt008	IMPACT_EXOME_ACCESS	Pre	Pre	Longitudinal	Lung Adenocarcinoma	Fusion	Somatic	KIF5B-RET	fusion	PR	-40	2	Yes	No	129	136	No				MET amp
EDD_ret_pt010	IMPACT_EXOME_ARCHER_ACCESS	Pre+Post	Pre	Baseline	Lung Adenocarcinoma	Fusion	Somatic	KIF5B-RET	fusion	PR	-76.923	7	Yes	No	888	755	Yes				
EDD_ret_pt011	IMPACT_EXOME_ARCHER_ACCESS	Pre	Pre	Baseline	Lung Adenocarcinoma	Fusion	Somatic	KIAA1468-RET	fusion	CR	-100	2	No	No	566		Yes				
EDD_ret_pt012	pIMPACT_ACCESS	Post		Baseline	Lung Adenocarcinoma	Fusion	Somatic	CLIP1-RET	fusion	PR	-76.19	3	Yes	No	429		No				
EDD_ret_pt013	IMPACT_EXOME_ACCESS	Pre	Pre	Longitudinal	Medullary Thyroid	Mutation	Somatic	D631_L633delinsE	CRD_indel_oncogenic	PR	-52.5	0	No	No	501	496	Yes				
EDD_ret_pt014	pIMPACT_ACCESS	Post		Longitudinal	Lung Adenocarcinoma	Fusion	Somatic	KIF5B-RET	fusion	PR	-47.059	2	Yes	No	420	332	No	RET_fusion			
EDD_ret_pt015	IMPACT_ACCESS	Pre		Longitudinal	Medullary Thyroid	Mutation	Somatic	C630G	CRD_oncogenic	PR	-35.088	2	Yes	Yes	566		Yes				
EDD_ret_pt016	pIMPACT_ACCESS	Post		Longitudinal	Lung Adenocarcinoma	Fusion	Somatic	KIF5B-RET	fusion	PD	-41.216	0	No	No	501	216	No	PI3K	PTEN E242*		
EDD_ret_pt017	IMPACT	Pre		REMOVED	Lung Adenocarcinoma	Fusion	Somatic	KIF5B-RET	fusion	PR	-35.345	0	No	No	649		Yes				
EDD_ret_pt018	IMPACT_EXOME_ACCESS	Pre	Pre	Baseline	Lung Adenocarcinoma	Fusion	Somatic	KIF5B-RET	fusion	PR	-55.978	3	Yes	Yes	641	677	No				
EDD_ret_pt019	ARCHER_ACCESS			Longitudinal	High-Grade Neuroendocrine Carcinoma of the Colon and Rectum	Fusion	Somatic	TAF3-RET	fusion	PD	3.057	4	No	No	25	21	No	PI3K	PIK3CA M1043V/PTEN Hotspots (4)		KRAS A59del/G12D
EDD_ret_pt020	IMPACT_EXOME_ACCESS	Pre+Post	Pre	Longitudinal	Medullary Thyroid	Mutation	Somatic	M918T	KD_oncogenic	PR	-61.538	0	No	No	497		Yes				
EDD_ret_pt021	IMPACT_EXOME_ACCESS	Pre	Pre	Baseline	Lung Adenocarcinoma	Fusion	Somatic	KIF5B-RET	fusion	PR	-32	3	No	Yes	830		Yes				
EDD_ret_pt023	ACCESS			Baseline	Lung Adenocarcinoma	Fusion	Somatic	KIF5B-RET	fusion	NE	-16.667	2	No	Yes	29		No	PI3K	AKT1 E17K		
EDD_ret_pt024	IMPACT_ARCHER_ACCESS	Pre		Baseline	Lung Adenocarcinoma	Fusion	Somatic	CCDC6-RET	fusion	PR	-78.689	1	No	No	523		Yes				
EDD_ret_pt025	IMPACT_EXOME_ARCHER_ACCESS	Pre+Post	Pre+Post	Longitudinal	Lung Adenocarcinoma	Fusion	Somatic	KIF5B-RET	fusion	SD	-1.408	1	No	No	167	105	No			PARK2-RAD21 fusion, MET amp	MET amp
EDD_ret_pt026	IMPACT_EXOME_ACCESS	Pre	Pre	Longitudinal	Medullary Thyroid	Mutation	Somatic	M918T	KD_oncogenic	SD	-20.513	1	Yes	No	405	409	No	PI3K	PIK3CA R93W		
EDD_ret_pt027	IMPACT_EXOME_ACCESS	Pre	Pre	Baseline	Lung Adenocarcinoma	Fusion	Somatic	KIF5B-RET	fusion	PR	-56.716	1	No	No	668	500	Yes				
EDD_ret_pt028	IMPACT_EXOME_ACCESS	Pre+Post	Pre+Post	Longitudinal	Lung Adenocarcinoma	Fusion	Somatic	KIF5B-RET	fusion	PR	-73.913	0	No	No	279	279	No				NRAS G13D
EDD_ret_pt029	IMPACT_ACCESS	Pre		Baseline	Papillary Thyroid	Fusion	Somatic	CCDC6-RET	fusion	PR	-40.625	4	No	No	454		No				
EDD_ret_pt030	IMPACT_EXOME_ARCHER_ACCESS	Pre	Pre	Baseline	Papillary Thyroid	Fusion	Somatic	RUFY3-RET	fusion	PR	-100	3	No	No	754		Yes				
EDD_ret_pt031	IMPACT_EXOME_ACCESS	Pre	Pre	Longitudinal	Medullary Thyroid	Mutation	Somatic	M918T	KD_oncogenic	PR	-55.245	1	No	No	860		Yes				
EDD_ret_pt032	IMPACT_EXOME_ARCHER_ACCESS	Pre	Pre	Baseline	Lung Adenosquamous	Fusion	Somatic	CCDC6-RET	fusion	CR	-100	0	No	No	523		Yes	PI3K	PTEN G165E		
EDD_ret_pt033	ACCESS			Baseline	Medullary Thyroid	Mutation	Germline	V804M	KD_pathogenic	PR	-81.25	0	No	Yes	545		Yes				
EDD_ret_pt034	IMPACT_EXOME_ACCESS	Pre	Pre	Baseline	Lung Adenocarcinoma	Fusion	Somatic	CCDC6-RET	fusion	SD	-26.19	1	Yes	No	629		Yes				
EDD_ret_pt035	IMPACT_ACCESS	Pre		Longitudinal	Medullary Thyroid	Mutation	Somatic	M918T	KD_oncogenic	PD	11.356	2	Yes	No	660	55	No	RET_mut	K666E		
EDD_ret_pt036	IMPACT_EXOME_ACCESS	Pre	Pre	Baseline	Lung Adenocarcinoma	Fusion	Somatic	KIF5B-RET	fusion	PR	-31.937	0	No	No	109	111	No				
EDD_ret_pt037	IMPACT_EXOME_ACCESS	Pre	Pre	Baseline	Lung Adenocarcinoma	Fusion	Somatic	CCDC6-RET	fusion	SD	NTL	4	Yes	No	607		Yes				
EDD_ret_pt038	ACCESS			Longitudinal	Medullary Thyroid	Mutation	Germline	K666N	TMD VUS	SD	-28.571	1	Yes	No	556		Yes	RET_mut	D898Y		
EDD_ret_pt039	EXOME_ARCHER_ACCESS		Pre	Baseline	Lung Adenocarcinoma	Fusion	Somatic	CCDC6-RET	fusion	SD	-28.947	2	Yes	Yes	555	497	No				
EDD_ret_pt040	ACCESS			Baseline	Lung Adenocarcinoma	Fusion	Somatic	CCDC6-RET	fusion	PR	-59.259	1	No	No	802		Yes				
EDD_ret_pt041	IMPACT_EXOME_ACCESS	Pre+Post	Pre+Post	Longitudinal	Lung Adenocarcinoma	Fusion	Somatic	CCDC6-RET	fusion	PR	-58.537	5	Yes	No	846	167	Yes			PTEN trunc, KMT2A-TMEM fusion, KMT2D trunc, FGFR1 amp	
EDD_ret_pt042	IMPACT_EXOME_ACCESS	Pre	Pre	Baseline	Lung Adenocarcinoma	Fusion	Somatic	KIF5B-RET	fusion	PR	-51.613	2	Yes	No	853		Yes				
EDD_ret_pt043	IMPACT_EXOME_ACCESS	Pre	Pre	Baseline	Papillary Thyroid	Fusion	Somatic	NCOA4-RET	fusion	PR	-54.545	4	No	No	830		Yes				
EDD_ret_pt044	IMPACT_EXOME_ACCESS	Pre	Pre	Longitudinal	Medullary Thyroid	Mutation	Germline	M918T	KD_pathogenic	PR	-100	3	Yes	No	515		Yes	RET_mut	G601E		
EDD_ret_pt045	IMPACT_EXOME_ACCESS	Pre	Pre	Longitudinal	Medullary Thyroid	Mutation	Somatic	E632_L633del	CRD_indel_oncogenic	PR	-45.631	1	Yes	Yes	853	833	Yes				
EDD_ret_pt047	ACCESS			Longitudinal	Combined Small Cell Lung Carcinoma	Fusion	Somatic	KIF5B-RET	fusion	PD	-69.504	1	No	No	445	280	No	PI3K	PIK3CA E545K		RET G810C
EDD_ret_pt048	IMPACT_EXOME_ACCESS	Pre+Post	Pre	Baseline	Lung Adenocarcinoma	Fusion	Somatic	KIF5B-RET	fusion	SD	-26.316	2	Yes	No	216	154	No	RET_fusion_mut		MYC amp	
EDD_ret_pt049	IMPACT_ACCESS	Pre+Post		Longitudinal	Poorly Differentiated Thyroid	Fusion	Somatic	CCDC6-RET	fusion	PD	-38.462	4	No	No	442	218	No			CDK4_amp	PIK3CA R115Q
EDD_ret_pt050	IMPACT_EXOME_ARCHER_ACCESS	Pre	Pre	Baseline	Lung Adenocarcinoma	Fusion	Somatic	KIF5B-RET	fusion	PR	-62.5	0	No	No	466		Yes				
EDD_ret_pt051	IMPACT_EXOME_ACCESS	Pre	Pre	Longitudinal	Medullary Thyroid	Mutation	Somatic	M918T	KD_oncogenic	SD	-12.5	3	Yes	No	465		Yes				
EDD_ret_pt052	IMPACT_EXOME_ARCHER_ACCESS	Pre	Pre	Baseline	Lung Adenocarcinoma	Fusion	Somatic	KIF5B-RET	fusion	PR	-45	0	No	No	447		Yes				
EDD_ret_pt053	IMPACT_EXOME_ACCESS	Pre	Pre	Longitudinal	Medullary Thyroid	Mutation	Somatic	M918T	KD_oncogenic	SD	NTL	2	Yes	No	439		Yes				
EDD_ret_pt054	pIMPACT_pEXOME_ARCHER_ACCESS	Post	Post	Longitudinal	Lung Adenocarcinoma	Fusion	Somatic	KIF5B-RET	fusion	SD	-22.086	1	Yes	No	223	223	No				RET G810S, L870F, KRAS G12A/R, HER2 amp, CTNNBL2-HER2 fusion
EDD_ret_pt055	ACCESS			Baseline	Medullary Thyroid	Mutation	Somatic	C634R	CRD_oncogenic	NE	No Assessments	2	Yes	Yes	5		No				
EDD_ret_pt056	IMPACT_EXOME_ACCESS	Pre	Pre	Baseline	Lung Adenocarcinoma	Fusion	Somatic	CCDC6-RET	fusion	PR	-100	2	Yes	Yes	377		Yes				
EDD_ret_pt057	IMPACT_EXOME	Pre	Pre	NONE	Lung Adenocarcinoma	Fusion	Somatic	KIF5B-RET	fusion	PR	-93.75	1	No	No	343		Yes	RET_mut	T1078M		
EDD_ret_pt058	IMPACT_EXOME_ARCHER_ACCESS	Pre+Post	Pre+Post	Longitudinal	Lung Adenocarcinoma	Fusion	Somatic	KIF5B-RET	fusion	SD	-19.209	0	No	No	71	95	No	PI3K	PIK3CA R115Q	KRAS G12V	KRAS G12V
EDD_ret_pt059	EXOME_ARCHER_ACCESS		Pre	Baseline	Non Langerhans Cell Histiocytosis	Fusion	Somatic	NCOA4-RET	fusion	CR	NTL	0	No	No	333		Yes				
EDD_ret_pt060	IMPACT_EXOME_ARCHER_ACCESS	Pre	Pre	Baseline	Lung Adenocarcinoma	Fusion	Somatic	KIF5B-RET	fusion	SD	-41.085	1	No	No	328		Yes				
EDD_ret_pt061	ACCESS			Baseline	Medullary Thyroid	Mutation	Germline	C634R	CRD_pathogenic	SD	-13.043	0	No	No	259		No				
EDD_ret_pt062	ACCESS			Baseline	Lung Adenocarcinoma	Fusion	Somatic	KIF5B-RET	fusion	SD	-15.909	0	No	Yes	299		Yes	PI3K	PTEN H61R		
EDD_ret_pt063	ACCESS			Baseline	Lung Adenocarcinoma	Fusion	Somatic	KIF5B-RET	fusion	SD	-17.391	1	No	No	293		Yes				
EDD_ret_pt064	IMPACT_EXOME_ACCESS	Pre	Pre	Baseline	Lung Adenocarcinoma	Fusion	Somatic	KIF5B-RET	fusion	PR	-48.958	0	No	No	286		Yes				
EDD_ret_pt065	EXOME_ARCHER_ACCESS		Pre	Baseline	Lung Adenocarcinoma	Fusion	Somatic	KIF5B-RET	fusion	PR	-55.738	0	No	No	278		Yes				
EDD_ret_pt066	IMPACT_ACCESS	Pre		Baseline	Medullary Thyroid	Mutation	Somatic	M918T	KD_oncogenic	SD	-38.235	1	No	Yes	272		Yes				
EDD_ret_pt067	ACCESS			Baseline	Lung Adenocarcinoma	Fusion	Somatic	KIF5B-RET	fusion	SD	-18.75	0	No	Yes	223	164	No	PI3K	PIK3CA R93W		
EDD_ret_pt069	IMPACT_EXOME_ACCESS	Pre	Pre	Baseline	Medullary Thyroid	Mutation	Germline	C634Y	CRD_pathogenic	PR	-100	0	No	No	255		Yes				
EDD_ret_pt070	IMPACT_EXOME_ARCHER_ACCESS	Pre	Pre	Longitudinal	Lung Adenocarcinoma	Fusion	Somatic	ERC1-RET	fusion	SD	-0.68	1	No	Yes	62	52	No	MAPK	KRAS G12V		KRAS G12V, NRAS Q61R
EDD_ret_pt071	IMPACT_EXOME_ACCESS	Pre	Pre	Baseline	Medullary Thyroid	Mutation	Somatic	C634R	CRD_oncogenic	PR	-86.275	3	Yes	No	244		Yes				
EDD_ret_pt072	IMPACT_ACCESS	Pre		Baseline	Lung Adenocarcinoma	Fusion	Somatic	KIF13A-RET	fusion	PR	-52.381	4	Yes	No	237		Yes	PI3K	PTEN splice		
EDD_ret_pt073	IMPACT_EXOME_ACCESS	Pre	Pre	Baseline	Medullary Thyroid	Mutation	Somatic	L629_L633del	CRD_indel_oncogenic	PR	-66.667	1	Yes	Yes	214		Yes				
EDD_ret_pt074	IMPACT_EXOME_ACCESS	Pre	Pre	Baseline	Papillary Thyroid	Fusion	Somatic	CCDC6-RET	fusion	SD	-11.111	4	No	No	202		Yes	PI3K	PIK3CA H1047Y		
EDD_ret_pt075	ACCESS			Baseline	Lung Adenocarcinoma	Fusion	Somatic	KIF5B-RET	fusion	PR	-67.391	1	No	Yes	193		Yes				
EDD_ret_pt076	ACCESS			Baseline	Lung Adenocarcinoma	Fusion	Somatic	CCDC6-RET	fusion	PR	-37.931	0	No	Yes	194		Yes				
EDD_ret_pt077	IMPACT_EXOME_ACCESS	Pre	Pre	Baseline	Medullary Thyroid	Mutation	Somatic	M918T	KD_oncogenic	PR	-19.048	1	Yes	Yes	160		Yes				

[a] NE, not evaluable

*Endpoint of either

Supplemental Table 3: Whole-exome sequencing

Study_ID	Tumor type	RET alteration	Tumor cov.	Normal cov.	Purity	Ploidy	TMB	WGD	MSIscor	% clonal mutations	Signature_1	Signature_2	Signature_3	Signature_4	Signature_5	Signature_6	Signature_7	Signature_8	Signature_9	Signature_10	Signature_11	Signature_12	Signature_13	Signature_14	Signature_15	Signature_16	Signature_17	Signature_18	Signature_19	Signature_20	Signature_21	Signature_22	Signature_23	Signature_24	Signature_25	Signature_26	Signature_27	Signature_28	Signature_29	Signature_30
EDD_ret_pt004	Papillary Thyroid	Fusion	136.9	82.6	0.47	1.7	1.4	No	0.1	41.2	0.00	0.06	0.00	0.00	0.23	0.16	0.00	0.00	0.10	0.06	0.00	0.00	0.10	0.00	0.14	0.00	0.00	0.00	0.00	0.00	0.00	0.00	0.00	0.04	0.00	0.00	0.00	0.00	0.00	0.11
EDD_ret_pt039	Lung Adenocarcinoma	Fusion	312.0	128.7	0.24	2.2	1.1	No	0.1	40.0	0.88	0.00	0.00	0.00	0.00	0.00	0.00	0.00	0.00	0.00	0.00	0.00	0.03	0.00	0.00	0.00	0.00	0.00	0.00	0.00	0.00	0.00	0.00	0.06	0.00	0.00	0.02	0.00	0.00	0.00
EDD_ret_pt013	Medullary Thyroid	Point	135.8	70.8	0.30	2.0	0.4	No	0.4	23.5	0.48	0.00	0.00	0.00	0.00	0.00	0.00	0.00	0.00	0.00	0.00	0.00	0.00	0.00	0.49	0.00	0.00	0.00	0.00	0.00	0.00	0.00	0.00	0.03	0.00	0.00	0.00	0.00	0.00	0.00
EDD_ret_pt045	Medullary Thyroid	Point	139.0	76.3	0.63	3.1	2.2	Yes	0.6	25.3	0.17	0.00	0.23	0.11	0.00	0.11	0.12	0.00	0.00	0.01	0.02	0.07	0.03	0.00	0.00	0.00	0.00	0.06	0.00	0.02	0.00	0.00	0.04	0.00	0.00	0.00	0.00	0.00	0.00	0.00
EDD_ret_pt008	Lung Adenocarcinoma	Fusion	173.4	78.4	0.91	2.1	2.4	No	1.3	50.6	0.22	0.26	0.00	0.00	0.00	0.00	0.04	0.00	0.04	0.00	0.00	0.00	0.25	0.00	0.07	0.00	0.00	0.00	0.00	0.00	0.00	0.01	0.00	0.10	0.00	0.00	0.00	0.01	0.00	0.00
EDD_ret_pt058	Lung Adenocarcinoma	Fusion	100.1	98.7	0.55	2.2	2.2	No	0.2	25.6	0.08	0.00	0.00	0.00	0.00	0.00	0.02	0.01	0.00	0.00	0.00	0.00	0.01	0.00	0.00	0.00	0.00	0.00	0.00	0.03	0.00	0.00	0.00	0.36	0.00	0.00	0.00	0.00	0.45	0.04
EDD_ret_pt044	Medullary Thyroid	Point	197.6	98.1	0.76	2.1	2.0	No	0.3	18.1	0.25	0.12	0.04	0.06	0.00	0.00	0.06	0.00	0.00	0.00	0.00	0.00	0.00	0.04	0.09	0.00	0.00	0.30	0.00	0.00	0.00	0.00	0.02	0.00	0.00	0.00	0.01	0.00	0.00	0.00
EDD_ret_pt042	Lung Adenocarcinoma	Fusion	301.3	79.0	0.20	1.8	1.1	No	0.7	42.5	0.44	0.00	0.11	0.00	0.00	0.00	0.04	0.00	0.00	0.00	0.08	0.00	0.04	0.00	0.04	0.00	0.00	0.00	0.00	0.00	0.00	0.00	0.00	0.00	0.00	0.10	0.01	0.00	0.14	0.00
EDD_ret_pt051	Medullary Thyroid	Point	160.1	81.8	0.20	2.0	1.2	No	0.6	0.0	0.13	0.02	0.06	0.00	0.00	0.34	0.00	0.00	0.00	0.00	0.03	0.00	0.06	0.00	0.15	0.00	0.00	0.00	0.00	0.00	0.00	0.00	0.00	0.08	0.00	0.00	0.00	0.06	0.07	0.00
EDD_ret_pt043	Papillary Thyroid	Fusion	176.3	85.0	0.79	2.1	0.8	No	0.0	0.0	0.31	0.00	0.12	0.00	0.00	0.00	0.00	0.00	0.00	0.00	0.00	0.00	0.04	0.00	0.28	0.00	0.00	0.00	0.00	0.00	0.00	0.06	0.02	0.17	0.00	0.00	0.00	0.01	0.00	0.00
EDD_ret_pt037	Lung Adenocarcinoma	Fusion	321.1	84.6	0.29	2.0	1.6	No	0.1	57.1	0.36	0.17	0.00	0.09	0.00	0.00	0.12	0.00	0.00	0.00	0.00	0.00	0.07	0.00	0.00	0.11	0.00	0.00	0.00	0.00	0.00	0.05	0.00	0.00	0.00	0.00	0.00	0.03	0.00	0.00
EDD_ret_pt071	Medullary Thyroid	Point	152.1	82.1	0.65	2.7	1.3	Yes	0.5	23.9	0.03	0.00	0.10	0.00	0.00	0.00	0.00	0.00	0.04	0.00	0.00	0.20	0.00	0.00	0.09	0.00	0.00	0.00	0.03	0.00	0.00	0.00	0.00	0.50	0.00	0.00	0.00	0.00	0.00	0.02
EDD_ret_pt025	Lung Adenocarcinoma	Fusion	131.1	78.2	0.20	2.1	2.4	No	0.0	36.4	0.15	0.05	0.06	0.00	0.00	0.21	0.02	0.00	0.00	0.00	0.00	0.00	0.33	0.00	0.04	0.00	0.00	0.00	0.01	0.00	0.01	0.00	0.00	0.09	0.01	0.00	0.02	0.00	0.00	0.00
EDD_ret_pt003	Lung Adenocarcinoma	Fusion	194.5	105.6	0.49	2.2	0.8	No	0.2	46.4	0.55	0.06	0.14	0.00	0.00	0.00	0.00	0.00	0.00	0.00	0.00	0.00	0.00	0.00	0.00	0.00	0.00	0.00	0.05	0.00	0.00	0.07	0.00	0.00	0.00	0.08	0.00	0.04	0.00	0.00
EDD_ret_pt034	Lung Adenocarcinoma	Fusion	185.4	84.8	0.34	2.1	1.1	No	0.0	35.7	0.30	0.08	0.00	0.00	0.00	0.16	0.00	0.00	0.00	0.06	0.06	0.03	0.00	0.04	0.00	0.00	0.04	0.15	0.00	0.04	0.00	0.00	0.00	0.00	0.00	0.00	0.04	0.00	0.00	0.00
EDD_ret_pt011	Lung Adenocarcinoma	Fusion	174.8	88.4	0.42	2.2	3.8	No	0.3	43.4	0.20	0.30	0.04	0.02	0.00	0.00	0.04	0.00	0.00	0.07	0.00	0.00	0.29	0.00	0.02	0.00	0.00	0.01	0.00	0.00	0.00	0.00	0.00	0.02	0.00	0.00	0.00	0.00	0.00	0.00
EDD_ret_pt031	Medullary Thyroid	Point	104.3	76.6	0.42	1.8	2.9	No	0.3	13.2	0.84	0.00	0.00	0.00	0.00	0.09	0.00	0.00	0.00	0.00	0.05	0.00	0.00	0.00	0.00	0.00	0.00	0.00	0.00	0.00	0.00	0.00	0.01	0.00	0.00	0.00	0.00	0.00	0.00	0.00
EDD_ret_pt069	Medullary Thyroid	Point	197.5	88.7	0.83	1.9	1.2	No	1.0	62.2	0.25	0.00	0.00	0.25	0.00	0.00	0.04	0.00	0.00	0.03	0.00	0.00	0.00	0.00	0.00	0.02	0.06	0.07	0.00	0.00	0.00	0.00	0.16	0.13	0.00	0.00	0.00	0.00	0.00	0.00
EDD_ret_pt010	Lung Adenocarcinoma	Fusion	286.1	83.3	0.21	1.9	1.7	No	0.4	62.1	0.31	0.08	0.24	0.00	0.00	0.00	0.12	0.00	0.04	0.00	0.00	0.00	0.04	0.00	0.07	0.00	0.00	0.00	0.00	0.00	0.00	0.03	0.00	0.05	0.00	0.00	0.00	0.00	0.00	0.02
EDD_ret_pt027	Lung Adenocarcinoma	Fusion	333.4	79.0	0.30	1.8	1.5	No	0.6	0.0	0.11	0.25	0.25	0.06	0.00	0.05	0.05	0.00	0.00	0.00	0.01	0.00	0.03	0.03	0.00	0.00	0.00	0.00	0.00	0.03	0.02	0.00	0.00	0.04	0.00	0.00	0.05	0.00	0.00	0.00
EDD_ret_pt070	Lung Adenocarcinoma	Fusion	342.9	98.1	0.32	1.7	3.4	No	0.4	30.5	0.02	0.29	0.12	0.00	0.00	0.00	0.18	0.00	0.00	0.05	0.00	0.00	0.20	0.00	0.02	0.00	0.00	0.00	0.00	0.00	0.00	0.00	0.00	0.05	0.07	0.00	0.00	0.00	0.00	0.00
EDD_ret_pt065	Lung Adenocarcinoma	Fusion	345.2	110.6	0.26	1.8	0.7	No	0.3	19.2	0.12	0.08	0.05	0.00	0.18	0.00	0.00	0.00	0.00	0.00	0.01	0.00	0.13	0.06	0.00	0.00	0.00	0.03	0.00	0.00	0.00	0.00	0.12	0.19	0.00	0.00	0.00	0.00	0.00	0.03
EDD_ret_pt036	Lung Adenocarcinoma	Fusion	173.0	71.5	0.50	1.9	1.5	No	0.7	67.3	0.24	0.29	0.19	0.04	0.00	0.01	0.00	0.00	0.00	0.00	0.00	0.00	0.12	0.00	0.00	0.00	0.00	0.03	0.00	0.02	0.01	0.00	0.00	0.00	0.00	0.00	0.00	0.00	0.03	0.03
EDD_ret_pt028	Lung Adenocarcinoma	Fusion	197.2	86.7	0.41	1.9	0.7	No	0.2	24.1	0.18	0.26	0.00	0.00	0.00	0.00	0.00	0.00	0.00	0.01	0.00	0.00	0.06	0.00	0.00	0.00	0.00	0.00	0.00	0.00	0.11	0.04	0.05	0.00	0.00	0.00	0.01	0.00	0.00	0.26
EDD_ret_pt021	Lung Adenocarcinoma	Fusion	185.1	87.1	0.27	2.0	0.7	No	0.0	8.0	0.09	0.12	0.03	0.16	0.00	0.32	0.00	0.00	0.00	0.05	0.00	0.00	0.05	0.00	0.00	0.00	0.00	0.00	0.04	0.00	0.00	0.12	0.01	0.00	0.00	0.00	0.01	0.00	0.00	0.00
EDD_ret_pt041	Lung Adenocarcinoma	Fusion	185.5	84.4	0.29	3.3	1.9	Yes	0.5	21.2	0.30	0.24	0.01	0.11	0.00	0.00	0.02	0.00	0.00	0.00	0.00	0.00	0.07	0.06	0.00	0.00	0.00	0.00	0.00	0.00	0.04	0.00	0.00	0.05	0.02	0.00	0.00	0.00	0.00	0.07
EDD_ret_pt059	Non-LCH	Fusion	303.9	96.7	0.20	2.0	0.2	No	0.5	0.0	0.00	0.00	0.19	0.00	0.00	0.27	0.00	0.00	0.00	0.00	0.00	0.01	0.00	0.00	0.00	0.00	0.00	0.00	0.00	0.00	0.00	0.00	0.12	0.10	0.00	0.00	0.00	0.15	0.15	0.00
EDD_ret_pt026	Medullary Thyroid	Point	182.1	85.7	0.70	2.0	1.2	No	0.2	75.0	0.00	0.00	0.00	0.00	0.00	0.00	0.17	0.00	0.05	0.05	0.05	0.00	0.02	0.00	0.18	0.00	0.04	0.00	0.00	0.22	0.00	0.00	0.00	0.21	0.00	0.00	0.00	0.00	0.00	0.01
EDD_ret_pt030	Papillary Thyroid	Fusion	276.3	82.1	0.20	2.0	1.7	No	0.7	0.0	0.12	0.23	0.00	0.16	0.04	0.00	0.05	0.00	0.00	0.00	0.00	0.00	0.27	0.00	0.00	0.00	0.00	0.03	0.00	0.00	0.09	0.00	0.00	0.00	0.00	0.00	0.00	0.00	0.00	0.00
EDD_ret_pt002	Lung Adenocarcinoma	Fusion	187.9	82.5	0.31	1.8	3.0	No	0.0	48.6	0.23	0.55	0.00	0.00	0.00	0.00	0.10	0.00	0.00	0.02	0.00	0.00	0.09	0.00	0.01	0.00	0.00	0.00	0.00	0.00	0.00	0.00	0.00	0.00	0.00	0.00	0.00	0.00	0.00	0.00
EDD_ret_pt064	Lung Adenocarcinoma	Fusion	310.3	133.2	0.29	2.9	2.1	Yes	0.2	35.1	0.38	0.02	0.00	0.00	0.00	0.00	0.11	0.00	0.00	0.03	0.00	0.00	0.23	0.00	0.00	0.03	0.00	0.00	0.00	0.04	0.00	0.02	0.00	0.01	0.00	0.00	0.02	0.00	0.09	0.00
EDD_ret_pt073	Medullary Thyroid	Point	177.4	135.6	0.78	3.7	1.6	Yes	0.6	35.0	0.07	0.05	0.00	0.26	0.00	0.00	0.00	0.03	0.00	0.02	0.22	0.00	0.00	0.07	0.00	0.12	0.02	0.00	0.02	0.00	0.00	0.00	0.00	0.09	0.00	0.01	0.02	0.00	0.00	0.00
EDD_ret_pt020	Medullary Thyroid	Point	146.5	71.6	0.88	1.9	1.0	No	0.6	47.2	0.37	0.00	0.00	0.12	0.19	0.00	0.00	0.00	0.00	0.00	0.00	0.00	0.01	0.00	0.00	0.00	0.00	0.00	0.00	0.00	0.00	0.00	0.12	0.08	0.00	0.06	0.00	0.07	0.00	0.00
EDD_ret_pt050	Lung Adenocarcinoma	Fusion	184.8	84.1	0.54	1.9	9.7	No	0.2	39.3	0.04	0.42	0.00	0.01	0.00	0.00	0.08	0.00	0.00	0.01	0.00	0.00	0.43	0.00	0.00	0.00	0.00	0.00	0.00	0.00	0.00	0.00	0.00	0.01	0.00	0.00	0.00	0.00	0.00	0.00
EDD_ret_pt018	Lung Adenocarcinoma	Fusion	193.0	86.1	0.77	3.2	1.7	Yes	2.3	24.2	0.39	0.14	0.00	0.00	0.00	0.00	0.03	0.02	0.00	0.00	0.03	0.13	0.15	0.00	0.00	0.03	0.00	0.00	0.01	0.00	0.00	0.00	0.00	0.03	0.00	0.00	0.00	0.04	0.00	0.00
EDD_ret_pt053	Medullary Thyroid	Point	143.4	64.7	0.80	1.8	3.5	No	0.7	10.2	0.14	0.00	0.34	0.05	0.00	0.32	0.00	0.00	0.00	0.00	0.01	0.00	0.01	0.00	0.04	0.00	0.00	0.00	0.00	0.00	0.00	0.00	0.03	0.04	0.00	0.00	0.00	0.00	0.00	0.02
EDD_ret_pt074	Papillary Thyroid	Fusion	367.1	126.5	0.88	2.0	0.3	No	0.7	54.5	0.01	0.00	0.00	0.00	0.09	0.00	0.00	0.20	0.00	0.00	0.00	0.00	0.04	0.00	0.14	0.00	0.05	0.09	0.00	0.00	0.05	0.00	0.00	0.00	0.00	0.00	0.00	0.00	0.00	0.34
EDD_ret_pt032	Lung Adenosquamous	Fusion	167.0	73.2	0.34	2.2	0.8	No	1.5	16.7	0.20	0.12	0.09	0.00	0.00	0.12	0.01	0.01	0.00	0.00	0.00	0.07	0.00	0.00	0.27	0.00	0.00	0.00	0.00	0.00	0.00	0.00	0.00	0.00	0.06	0.04	0.01	0.00	0.00	0.00
EDD_ret_pt077	Medullary Thyroid	Point	171.9	99.6	0.20	2.0	0.4	No	0.1	0.0	0.34	0.00	0.06	0.15	0.00	0.00	0.06	0.00	0.00	0.00	0.00	0.00	0.00	0.00	0.34	0.00	0.00	0.00	0.00	0.00	0.00	0.00	0.00	0.00	0.00	0.01	0.00	0.04	0.00	0.00
EDD_ret_pt048	Lung Adenocarcinoma	Fusion	100.3	63.7	0.45	3.8	4.8	Yes	0.1	10.8	0.44	0.01	0.14	0.00	0.00	0.07	0.01	0.00	0.00	0.00	0.00	0.00	0.07	0.00	0.00	0.00	0.03	0.00	0.08	0.00	0.00	0.00	0.00	0.16	0.00	0.00	0.00	0.00	0.00	0.00
EDD_ret_pt056	Lung Adenocarcinoma	Fusion	280.2	71.9	0.30	2.2	0.9	No	0.4	12.5	0.45	0.01	0.19	0.00	0.00	0.00	0.01	0.00	0.00	0.00	0.00	0.00	0.00	0.00	0.00	0.00	0.00	0.00	0.00	0.00	0.00	0.04	0.00	0.00	0.00	0.06	0.22	0.00	0.03	0.00
EDD_ret_pt060	Lung Adenocarcinoma	Fusion	163.6	108.5	0.32	2.2	1.6	Yes	0.0	9.7	0.27	0.22	0.00	0.16	0.00	0.06	0.10	0.00	0.00	0.00	0.00	0.00	0.09	0.00	0.00	0.00	0.00	0.00	0.00	0.00	0.00	0.00	0.00	0.00	0.03	0.00	0.00	0.00	0.04	0.03
EDD_ret_pt057	Lung Adenocarcinoma	Fusion	302.4	109.2	0.23	1.9	1.9	No	0.1	11.1	0.54	0.00	0.02	0.10	0.00	0.00	0.13	0.00	0.00	0.00	0.00	0.01	0.01	0.00	0.00	0.07	0.00	0.00	0.00	0.00	0.00	0.00	0.08	0.02	0.00	0.00	0.02	0.00	0.00	0.00
EDD_ret_pt052	Lung Adenocarcinoma	Fusion	137.1	87.5	0.24	1.7	1.1	No	0.1	26.2	0.45	0.04	0.00	0.00	0.00	0.06	0.02	0.00	0.00	0.00	0.04	0.00	0.09	0.00	0.11	0.00	0.00	0.07	0.00	0.00	0.00	0.03	0.00	0.00	0.00	0.09	0.00	0.00	0.00	0.00

Supplemental Table 4: Annotation of prior MKI response. The details of prior MKI therapy for all patients with an additional RET alteration detected at baseline prior to selpercatinib treatment.

Patient	Line of therapy	Therapy	Duration (mo)	Reason for discontinuation	Enrolling RET	Additional RET
pt006	1	Vandetanib	11	Progression	M918T	V804M
pt006	2	Cabozantinib	2.5	Progression		
pt006	3	Capecitabine, temozolomide	11	Progression		
pt035	1	Carboplatin+paclitaxel	2	Completed therapy	M918T	K666E
pt035	2	Vandetanib	1	Progression		
pt035	3	-	-	-		
pt038	1	Vandetanib	3	Drug intolerance	K666N[a]	D898Y
pt038	2	-	-	-		
pt038	3	-	-	-		
pt044	1	Vandetanib	9.5	Progression	M918T[a]	G601E
pt044	2	Cabozantinib	3	Progression		
pt044	3	Pembrolizumab	4.5	Progression		
pt057	1	Cisplatin+vinorelbine	Adjuvant	Adjuvant	KIF5B-RET	T1078M
pt057	2	Carboplatin+paclitaxel	Unknown	Completed therapy		
pt057	3	Pemetrexed+brain irradiation	Unknown	Completed therapy		

[a] Indicates germline

Supplemental Table 5: Paired pre- and post-progression tissue and plasma results

Patient	Tumor Type	RET Alteration	Tissue Sequencing	Plasma Sequencing	Resistance Type	Resistance Results
pt019	HGNEC	TAF3-RET	None	Multiple	Primary	*KRAS* G12D
pt070	Lung Adeno	ERC1-RET	Pre-tx	Multiple	Primary	*KRAS* G12V
pt058	Lung Adeno	KIF5B-RET	Pre-, Post-tx	Multiple	Acquired	*KRAS* G12V
pt008	Lung Adeno	KIF5B-RET	Pre-tx	Multiple	Acquired	*MET* amp
pt025	Lung Adeno	KIF5B-RET	Pre-, Post-tx	Multiple	Acquired	*MET* amp (fc 2.1), BRAF D584N
pt054	Lung Adeno	KIF5B-RET	None	Multiple	Acquired	*RET* G810S/L870F, *KRAS* G12A/R
pt028	Lung Adeno	KIF5B-RET	Pre-, Post-tx	Multiple	Acquired	*NRAS* G13D
pt041	Lung Adeno	CCDC6-RET	Pre-, Post-tx	Multiple	Oligo-progression	*FGFR1* amp (fc 2.4)
pt047	LCNEC	KIF5B-RET	None	Multiple	Acquired	*RET* G810C
pt006	MTC	M918T, V804M	Pre-, Post-tx	Multiple	Acquired	*RET* Y806C, *KRAS* G12D/13D
pt049	PTC	CCDC6-RET	Pre-, Post-tx	Multiple	Oligo-progression	*PIK3CA* R115Q
pt048	Lung Adeno	KIF5B-RET	Pre-, Post-tx	Baseline	None	NA
pt004	PTC	CCDC6-RET	Pre-tx	Multiple	None	NA
pt026	MTC	M918T	Pre-tx	Multiple	None	NA
pt014	Lung Adeno	KIF5B-RET	None	Multiple	None	NA
pt016	Lung Adeno	KIF5B-RET	None	Multiple	None	NA
pt010	Lung Adeno	KIF5B-RET	Pre-, Post-tx	Baseline	None	NA
pt001	Lung Adeno	CCDC6-RET	None	Multiple	None	NA

HGNEC: High-grade neuroendocrine carcinoma of the colon and rectum, LCNEC: large cell neuroendocrine carcinoma of the lung,, MTC: medullary thyroid cancer, PTC: papillary thyroid cancer, Pre-tx: Pre-treatment, Post-tx: Post-treatment, fc: fold change
