# Supplementary material for: The evolution of RET inhibitor resistance in RET-driven lung and thyroid cancers
Source: Nat Commun. 2022 Mar 18;13:1450. doi: 10.1038/s41467-022-28848-x (PMC8933489; doi:10.1038/s41467-022-28848-x)
Supplement: Supplementary file 3 — Reporting Summary [file 41467_2022_28848_MOESM3_ESM.pdf]

nature portfolio

Corresponding author(s): Alexander Drilon

Last updated by author(s): Jan 5, 2022



# Reporting Summary

Nature Portfolio wishes to improve the reproducibility of the work that we publish. This form provides structure for consistency and transparency in reporting. For further information on Nature Portfolio policies, see our Editorial Policies and the Editorial Policy Checklist.

## Statistics

For all statistical analyses, confirm that the following items are present in the figure legend, table legend, main text, or Methods section.

| n/a | Confirmed | |
|---|---|---|
| ☐ | ☒ | The exact sample size (*n*) for each experimental group/condition, given as a discrete number and unit of measurement |
| ☐ | ☒ | A statement on whether measurements were taken from distinct samples or whether the same sample was measured repeatedly |
| ☐ | ☒ | The statistical test(s) used AND whether they are one- or two-sided<br>*Only common tests should be described solely by name; describe more complex techniques in the Methods section.* |
| ☐ | ☒ | A description of all covariates tested |
| ☒ | ☐ | A description of any assumptions or corrections, such as tests of normality and adjustment for multiple comparisons |
| ☐ | ☒ | A full description of the statistical parameters including central tendency (e.g. means) or other basic estimates (e.g. regression coefficient) AND variation (e.g. standard deviation) or associated estimates of uncertainty (e.g. confidence intervals) |
| ☐ | ☒ | For null hypothesis testing, the test statistic (e.g. *F*, *t*, *r*) with confidence intervals, effect sizes, degrees of freedom and *P* value noted<br>*Give P values as exact values whenever suitable.* |
| ☒ | ☐ | For Bayesian analysis, information on the choice of priors and Markov chain Monte Carlo settings |
| ☒ | ☐ | For hierarchical and complex designs, identification of the appropriate level for tests and full reporting of outcomes |
| ☒ | ☐ | Estimates of effect sizes (e.g. Cohen's *d*, Pearson's *r*), indicating how they were calculated |

*Our web collection on statistics for biologists contains articles on many of the points above.*

## Software and code

Policy information about availability of computer code

| | |
|---|---|
| Data collection | No software was used. |
| Data analysis | WES data were processed and analyzed using the Tempo pipeline (v1.3, https://ccstempo.netlify.app/). Demultiplexed FASTQ files were aligned to the b37 assembly of the human reference genome from the GATK bundle using BWA mem (v0.7.17). Aligned reads were converted and sorted into BAM files using samtools (v1.9) and marked for PCR duplicates using GATK MarkDuplicates (v3.8-1). PCR replicates were collapsed into consensus sequences using the in-house Marianas algorithm (https://github.com/mskcc/Marianas).Somatic mutations (SNVs and small indels) were called in tumor-normal pairs using MuTect2 (v4.1.0.0) and Strelka2 (v2.9.10), and structural variants were detected using Delly (v0.8.2) and Manta (v1.5.0). Tumor purity and ploidy as well as total and allele-specific copy number using the FACETS algorithm (v0.5.14, http://github.com/mskcc/facets). Consensus reads with representation from both strands of the original cfDNA duplex were used for de novo variant calling using VarDict (v1.5.1). De novo RET fusion calling was performed using Manta (v1.5.0). MSI status was assessed using MSIsensor (v0.5). All statistical analysis and figures were generated using R software. Source code for these analyses is available at http://github.com/taylor-lab/RET. |

For manuscripts utilizing custom algorithms or software that are central to the research but not yet described in published literature, software must be made available to editors and reviewers. We strongly encourage code deposition in a community repository (e.g. GitHub). See the Nature Portfolio guidelines for submitting code & software for further information.

## Data

Policy information about availability of data

All manuscripts must include a data availability statement. This statement should provide the following information, where applicable:

- Accession codes, unique identifiers, or web links for publicly available datasets
- A description of any restrictions on data availability
- For clinical datasets or third party data, please ensure that the statement adheres to our policy

The whole exome sequencing data are desposited at dbGAP under the following accession number: phs001783. Patient level clinical and genomic data from the study cohort is available through the cBioortal or Cancer Genomics: www.cbioportal.org/study/summary/?id=ret_mskcc_2020.
All other genomic and clinical data accompanies the manuscript and is available as Extended Data and Supplmentary Information.

# Field-specific reporting

Please select the one below that is the best fit for your research. If you are not sure, read the appropriate sections before making your selection.

☒ Life sciences ☐ Behavioural & social sciences ☐ Ecological, evolutionary & environmental sciences

For a reference copy of the document with all sections, see nature.com/documents/nr-reporting-summary-flat.pdf

# Life sciences study design

All studies must disclose on these points even when the disclosure is negative.

| | |
|---|---|
| Sample size | The patients in this cohort had a confirmed RET activating fusion or mutation and were subsequently treated with selpercatinib on the phse 1/2 LIBRETTO-001 trial at Memorial Sloan Kettering Cancer Center (ClinicalTrials.gov #NCT03157128). One patient in the study cohort was treated on a single patient-use protocol due to the presence of leptomeningeal disease. We sought to characterize the molecular determinants of response and resistance to selective RET inhibition in RET altered solid tumors. Data from all patients enrolled on the clinical trial at MSKCC was analyzed. No sample size calculation was performed with regards to this analysis. |
| Data exclusions | Two patients were excluded from analysis as they were lost to follow up. |
| Replication | Cell culture experiments were repeated as described in figure legends Figure 3, n = 2; Extended Data Figure 8, n = 2. All attempts to confirm replication were successful, and standard best laboratory practices were maintained to ensure data reproducibility. |
| Randomization | No randomization of data was performed. This is not applicable to either the patients on this trial (who all received study drug) or the experiments performed (in which there were clear control and experimental conditions). |
| Blinding | No blinding of data was performed. All patients on this clinical trial received study drug as is typical of an early phase clinical trial. The clinical data obtained and analyzed here therefore come wholly from patients who received study drug. |

# Reporting for specific materials, systems and methods

We require information from authors about some types of materials, experimental systems and methods used in many studies. Here, indicate whether each material, system or method listed is relevant to your study. If you are not sure if a list item applies to your research, read the appropriate section before selecting a response.

## Materials & experimental systems

| n/a | Involved in the study | |
|---|---|---|
| ☐ | ☒ | Antibodies |
| ☐ | ☒ | Eukaryotic cell lines |
| ☒ | ☐ | Palaeontology and archaeology |
| ☒ | ☐ | Animals and other organisms |
| ☐ | ☒ | Human research participants |
| ☐ | ☒ | Clinical data |
| ☒ | ☐ | Dual use research of concern |

## Methods

| n/a | Involved in the study | |
|---|---|---|
| ☒ | ☐ | ChIP-seq |
| ☒ | ☐ | Flow cytometry |
| ☒ | ☐ | MRI-based neuroimaging |

## Antibodies

| | |
|---|---|
| Antibodies used | The following antibodies were used: RET mAB (CST, #14556), pRET Y905 (CST, #3221), ERK (CST #9102S), pERK T202/Y204 (CST, #4370S), KRAS *(LBio, #LS-C175665-100) and actin (CST, #4970S), CD56 (clone MRQ42, Cell Marque), Chromagrannin A (clone LK2H10, Ventana), Ki-67 (clone MIB1, Dako), Synaptophysin (clone SNP88, Bio Genex). |

| | |
|---|---|
| Validation | Ret (E1N8X) XP® Rabbit mAb validated on extracts from various cell lines including TT and THP-1 cells (Oncogene. 1999 Jul 1;18 (26):3919-22). pRET Y905 (CST, #3221) validated against recombinant human GST-Ret (His658-Asp1110) and on extracts from the TT human medullary thyroid carcinoma cell line (Pharmaceuticals (Basel). 2021 Jan 6;14(1):38). pERK T202/Y204 (CST) validated on extracts from COS cells, untreated or treated with either U0126 #9903 (10 µM for 1h) or TPA #4174 (200 nM for 10 m; Nat Med. 2019 Sep;25(9):1422-1427). ERK (CST,#9102S) validated on extracts from HeLa cells transfected with 100 nM control siRNA #6201 (-) or p44 MAPK (Erk1) siRNA (Nat Med. 2019 Sep;25(9):1422-1427). Actin (CST, #4970S) validated against recombinant Actin isoforms (Nat Med. 2019 Sep;25(9):1422-1427). KRAS (LSBio, #LS-C175665-100) validated in the following manuscript: Nature communications. 2017 July;8:16111. |

## Eukaryotic cell lines

Policy information about cell lines

| | |
|---|---|
| Cell line source(s) | MZ-CRC-1 Medullary Thyroid Carcinoma cells were obtained from Dr. Fagin laboratory. HEK 293T cells were purchased from ATCC. |
| Authentication | The cell lines were not authenticated. |
| Mycoplasma contamination | Cells were routinely tested for mycoplasma and they tested mycoplasma negative. |
| Commonly misidentified lines (See ICLAC register) | No commonly misidentified cell lines were used in this study. |

## Human research participants

Policy information about studies involving human research participants

| | |
|---|---|
| Population characteristics | Median age at enrollment 62.5 years.<br>Male/Female: 53%/47%.<br>Extended data available in extended data tables 1-3. |
| Recruitment | Patients with confirmed RET activating fusion or mutation were recruited by the Early Drug Development Group at Memorial Sloan Ketttering, qualified for treatment based on screening guidelines, and were subsequently treated with selpercatinib. Patients qualified for enrollment if they harbored activating RET alterations and were recruited either using automated software identifying patients with these mutations on internal MSKCC sequencing platforms, or by outside referral. Patients then needed to meet strict inclusion criteria to be enrolled. All of the above are efforts to reduce any possible self selection biases in patient accrual. |
| Ethics oversight | This protocol was approved by the Institutional Review Board at Memorial Sloan Kettering Cancer Center. |

Note that full information on the approval of the study protocol must also be provided in the manuscript.

## Clinical data

Policy information about clinical studies

All manuscripts should comply with the ICMJEguidelines for publication of clinical research and a completedCONSORT checklist must be included with all submissions.

| | |
|---|---|
| Clinical trial registration | NCT03157128 |
| Study protocol | The study protocol can be accessed as supplemental information related to the primary clinical manscript (Drilon et al, NEJM 2020), located here: https://www.nejm.org/doi/suppl/10.1056/NEJMoa2005653/suppl_file/nejmoa2005653_protocol.pdf |
| Data collection | Included in the study cohort are patients enrolled on this trial at Memorial Sloan Kettering between May 2017 and clinical data freeze for analysis in December 2019. Trial data were collected in outpatient clinic and radiographic facilities at Memorial Sloan Kettering Cancer Center. |
| Outcomes | Outcome measures included ORR (overall response rate) by RECIST which were provided by the sponsor of this clinical trial, Loxo Oncology. RECIST was calculated using RECIST 1.1 (Response Evaluation in Solid Tumors Version 1.1) criteria. PFS (progression free survival) was defined as radiographic progression or death, for which centrally sources data from Loxo Oncology was used to calculate this outcome measure. Radiographic progression was assessed by serial imaging scans as detailed in the study protocol above. |
