# Supplementary material for: The evolution of RET inhibitor resistance in RET-driven lung and thyroid cancers
Source: Nat Commun. 2022 Mar 18;13:1450. doi: 10.1038/s41467-022-28848-x (PMC8933489; doi:10.1038/s41467-022-28848-x)

Refers to Figure 3B

Precision Plus Protein™
All Blue Prestained
Protein Standards

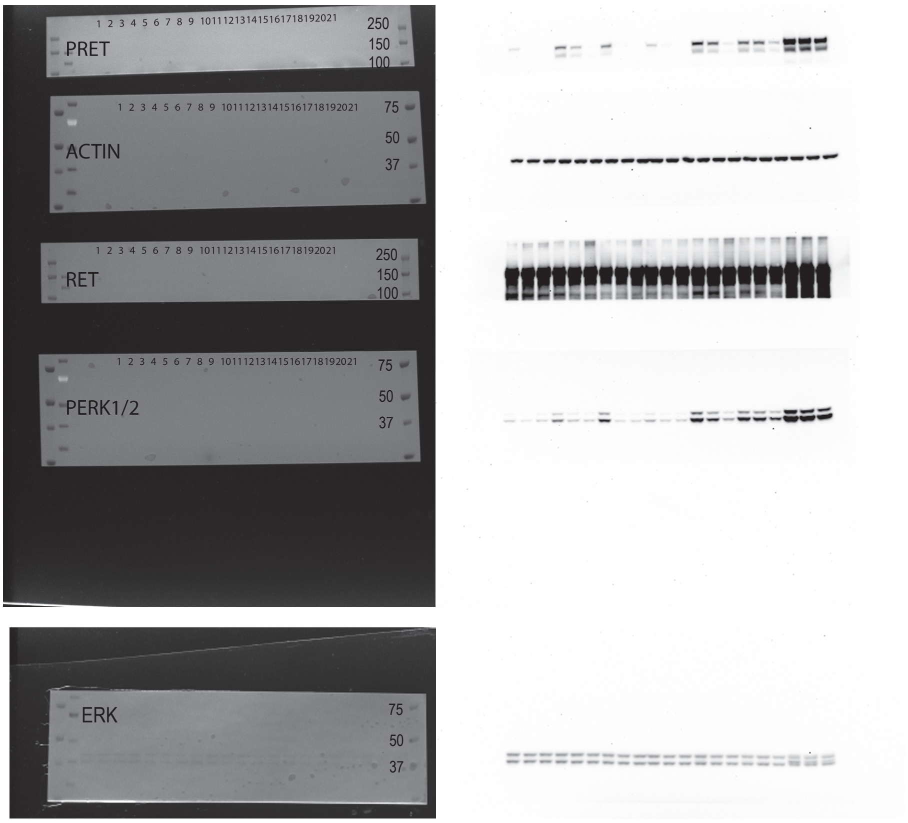

Refers to Extended Data Figure 8

Precision Plus Protein™
All Blue Prestained
Protein Standards

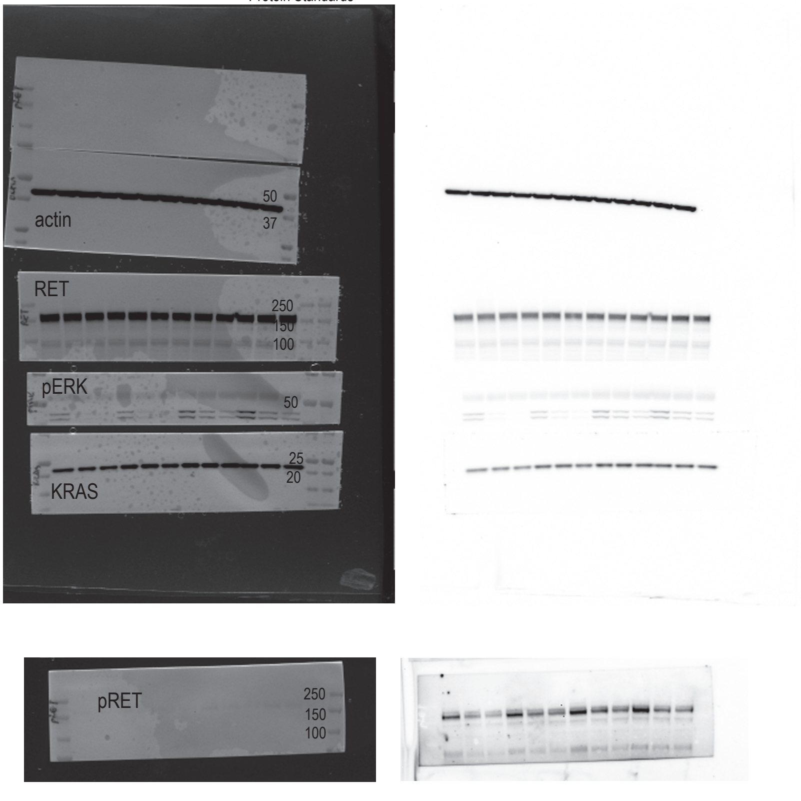

Refers to Extended Data Figure 8

Precision Plus Protein™
All Blue Prestained
Protein Standards

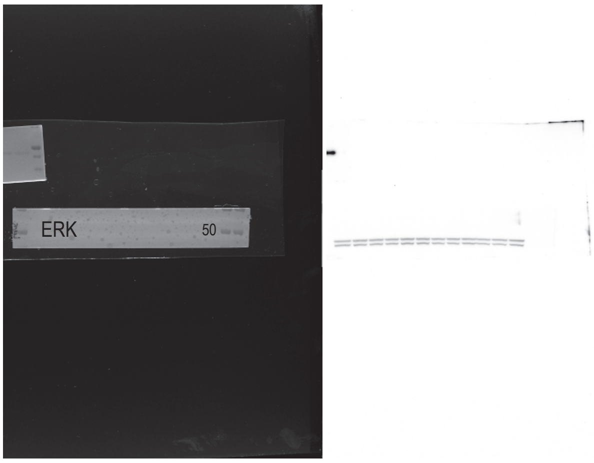

Supplement: Supplementary file 4 — Source Data [file 41467_2022_28848_MOESM4_ESM.pdf]
